# Congenital Hyperinsulinism in Neonates: Diagnostic Challenges and Management in Two Cases With KCNJ11 and ABCC8 Mutation

**DOI:** 10.7759/cureus.103829

**Published:** 2026-02-18

**Authors:** Adele Figuccia, Giuliana Vitaliti, Claudia Avanti, Luca Di Bella, Salvino Marcello Vitaliti

**Affiliations:** 1 Department of Health Promotion, Mother and Child Care, Internal Medicine and Medical Specialities, University of Palermo, Palermo, ITA; 2 Department of Neonatology, ARNAS Civico, Palermo, ITA

**Keywords:** congenital hyperinsulinism, diazoxide, hypoglycemia, neonatal sepsis, neonate

## Abstract

We report two cases of persistent neonatal hypoglycemia associated with mutations in the *KCNJ11* and *ABCC8* genes, encoding the Kir6.2 and SUR1 subunits of the adenosine triphosphate-sensitive potassium channel. In both cases, symptoms were non-specific and initially attributed to other conditions, including suspected infection. Standard treatment with enteral and parenteral glucose infusions failed to restore euglycemia. Diazoxide was administered without a clinical response. Both patients were then treated with octreotide, resulting in stabilization of glycemic levels. Genetic testing confirmed the presence of pathogenic variants consistent with congenital hyperinsulinism. Early identification and targeted management were crucial to achieving metabolic control.

## Introduction

Neonatal hyperinsulinemic hypoglycemia (HH) is a leading cause of neonatal care admission [1]. In 1988, through a multicenter nutritional study, Lucas et al. [2] originally proposed <47 mg/dL as the critical glucose concentration associated with adverse outcomes. However, current guidelines emphasize that there is no single universally accepted threshold. The American Academy of Pediatrics (AAP) operational thresholds vary according to postnatal age (25-40 mg/dL in the first 4 hours, 35-45 mg/dL from 4 to 24 hours of life, and 45 mg/dL after 24 hours of life) [3], while the Pediatric Endocrine Society (PES) recommends maintaining plasma glucose concentrations of >50 mg/dL during the first 48 hours and >60 mg/dL thereafter in high-risk neonates without suspected congenital hypoglycemic disorders. In cases of persistent or congenital hypoglycemia, higher targets (≥70 mg/dL) are suggested [4]. 

During the transition from intrauterine to extrauterine life, within the first 24-48 hours, healthy neonates typically exhibit lower blood glucose levels compared with later in life. It is important to distinguish this normal transitional alteration in glucose regulation from hypoglycemia that persists or appears after the first three days of life. When hypoglycemia is prolonged, it is most commonly linked to underlying congenital or genetic abnormalities affecting insulin secretion, deficiencies in cortisol and/or growth hormone, or metabolic disorders involving glucose, glycogen, or fatty acid processing [4].

The most common cause of persistent hypoglycemia in infants and children is congenital hyperinsulinism (HI) [5]. In this condition, inappropriate insulin secretion is due to a primary defect in the pancreatic beta cells. The inhibition of glycogenolysis and gluconeogenesis, combined with the stimulation of glucose uptake by muscle and adipose tissue due to insulin, results in reduced glycemia. This mechanism supports the rationale behind several important aspects of the clinical management of HI, such as the need for an elevated glucose infusion rate to avoid hypoglycemia and the typically excellent response to exogenous glucagon administration in case of hypoglycemia, as glucagon acts in opposition to insulin, promoting glycogenolysis and gluconeogenesis. At the same time, insulin, in order to protect the brain from neuroglucopenia, inhibits lipolysis and prevents compensatory ketogenesis [6].

Neonatal-onset HI may arise from either genetic abnormalities or acquired conditions. Acquired forms are commonly associated with perinatal factors such as maternal diabetes, perinatal stress, birth asphyxia, intrauterine growth restriction, maternal medication exposure, or excessive maternal glucose administration during labor and delivery [7].

Although mutations in genes regulating insulin secretion represent the primary cause of genetic forms of congenital HI, other etiologies include overgrowth syndromes, such as Beckwith-Wiedemann syndrome, and metabolic conditions, such as congenital disorders of glycosylation [8].

Herein, we present two cases of infants diagnosed with HI, each with a different clinical course. In the case of the first patient, persistent neonatal hypoglycemia (due to congenital HI from a K-channel inactivating mutation, *ABCC8*) overlaps with neonatal sepsis. This case highlights the importance of accurate differential diagnosis and prompt therapeutic intervention, as complex conditions such as sepsis may obscure early recognition. Early treatment is crucial to prevent long-term sequelae such as cerebral palsy, seizure disorders, and neurodevelopmental impairment [8].

## Case presentation

Case A

Our patient was born at the 39th week of gestation by spontaneous delivery. The maternal and pregnancy history includes grade II obesity and hypoglycemic episodes. During labor, fetal tachycardia and maternal hyperpyrexia occurred. At birth, the amniotic fluid was stained with meconium, and there was a nuchal cord. The newborn was hypotonic, hyporeactive, with absent crying and a heart rate of <100 bpm. Neonatal resuscitation was performed with Neopuff for about one minute, resulting in an improvement in vital signs, tone, and reactivity. Apgar scores were 1°minute 3, 5°minute 8, and 10°minute 9; weight was 3080 g (21st percentile), length was 50 cm (42nd percentile), and cranial circumference was 34.5 cm (44th percentile). Upon arrival in the intensive room, the newborn was placed in an incubator with continuous monitoring of vital parameters. The patient appeared distressed, with expiratory grunting (SpO_2_ 98% in air and heart rate 170 bpm) and mild hypertonia in all four limbs and tremors in the upper limbs. His Sarnat and Sarnat score was 1. Due to perinatal and maternal history, cultural examinations (blood, cerebrospinal fluid, urine, and bronchoaspirate) and hematological examinations were performed, which showed an increase in inflammatory markers. For this reason, and due to the finding of a serum glucose level of 47 mg/dL, a peripheral venous access was obtained and partial parenteral nutrition with glucose solution at 8% was initiated. Broad-spectrum antibiotic therapy with ampicillin and amikacin was also initiated. During the second day of life, the patient experienced rapid clinical deterioration with signs of autonomic nervous system dysfunction, including systemic hypotension, mottled skin with prolonged capillary refill time (three seconds), acrocyanosis, erythematous macular skin lesions, and thermoregulatory instability, together with a further increase in inflammatory markers. 

Transthoracic echocardiography subsequently demonstrated biventricular hypokinesia with reduced ejection fraction (approximately 40%) in the absence of structural congenital heart disease. Therefore, central venous access was obtained through umbilical vein catheterization, and therapy with inotropes and diuretics was initiated. Additionally, the antibiotic therapy was changed to meropenem and vancomycin. Transfontanellar ultrasound showed findings consistent with hypoxic-ischemic encephalopathy (marked periventricular hyper echogenicity with a focal appearance in the anterior coronal and right periventricular areas). On the third day of life, the onset of clonic seizures in the left hemibody occurred in the absence of fever and/or glucose-electrolyte imbalances. After excluding causes (hemorrhagic and ischemic) from the central nervous system, and after confirming the epileptic nature of the seizures through video EEG, intravenous therapy with phenobarbital was initiated. This therapy allowed for an improvement in the clinical conditions. On the seventh day of hospitalizations, due to the persistence of frequent hypoglycemic episodes (confirmed by low capillary blood glucose levels, such as 47 mg/dL), despite continuous high glucose infusion (10% glucose solution, with an infusion rate of 15 mg/kg/min), serial serum insulin and C-peptide levels were measured and HI was confirmed (Table 1). 

**Table 1:** Insulin and glucose levels confirming the diagnosis of hypoglycemia due to hyperinsulinism. Reference values are reported in the last row.

Case	Glucose (mg/dL)	Insulin (μU/mL)
Case 1	47	193
Case 2	27	33.6
Reference values	50-90	2.60-24.90

Oral diazoxide therapy (the maximum dose reached was 15 mg/kg/day) was started. On the 12th day of life, due to the persistence of severe hypoglycemia and the need for high glucose infusion rates, diazoxide was replaced with subcutaneous octreotide (initiated at 1 mcg/kg every 6 hours).

The following investigations were carried out: abdominal ultrasound (homogeneous pancreas), brain MRI, abdominal MRI with and without contrast (increased enhancement of the uncinate process of the pancreas in the arterial and portal phases), and (^18^F)-dihydroxyphenylalanine (^18^F-DOPA) PET/MRI (diffuse form of pancreatic beta-cell hyperplasia, increased pancreatic activity).

Next-generation sequencing (NGS) panel for HI provided a diagnosis of congenital HI with a mutation in the *KCNJ11* gene in homozygosity, encoding the subunits of the K^+^-adenosine triphosphate (ATP) channel, which is involved in the regulation of glucose-induced insulin secretion. The patient was discharged at three and a half months of life in good glycemic control with subcutaneous octreotide therapy (10 mcg/kg every six hours) and remained on antiepileptic treatment with phenobarbital. Serial EEGs demonstrated asymmetric theta-delta activity with greater left hemispheric involvement, which progressively improved over time, and phenobarbital therapy was adjusted accordingly.

Case B

Our patient was born at 36 + 5 weeks of gestation from a gravida 3, para 2 (G3P2) pregnancy that proceeded uneventfully and resulted in spontaneous delivery. The family history includes a paternal history of neonatal hypoglycemia, which was resolved spontaneously. At birth, the Apgar scores were 1°minute 10 and 5°minute 10, weight was 3390 g (89th percentile), length was 49 cm (62nd percentile), and cranial circumference was 34 cm (61st percentile). Clinical examination was normal, except for a suspected fracture of the left clavicle. At birth, a condition of hypoglycemia has been detected (glucose level of 27 mg/dL), which was unresponsive to enteral feeding and resolved after parenteral supplementation with a glucose solution (glucose intake was approximately 8 g/kg/day). After progressive reduction until the suspension of intravenous glucose supplementation, on the eighth day of recovery, the patient was discharged. During the post-recovery follow-up, due to the finding of an altered glucose level below 35 mg/dL, the neonate was admitted to our neonatal intensive care unit. He presented in good clinical condition, with a birth weight of 3490 g (81st percentile) and stable vital signs. The newborn was subjected to clinical and laboratory monitoring. Due to the finding of values initially at the lower limits, a continuous infusion of 8% glucose solution was administered, corresponding to an initial glucose infusion rate of 15 mg/kg/min, and later, on the fourth day of hospitalization, it was replaced with a 10% glucose solution, with a similar glucose supply. Laboratory tests showed low blood glucose levels associated with elevated insulin levels (Table 1).

A continuous glucose monitoring (CGM) system was implanted. The CGM revealed reduced glucose values both pre- and postprandial. On the ninth day of hospitalization, the newborn started therapy with diazoxide, progressively increased due to lack of benefit (maximum dose reached: 15 mg/kg/day). Due to the continued lack of response to diazoxide, it was then replaced with octreotide, initially at a dose of 5 mcg/kg every six hours.

During hospitalization, hematochemical tests (to exclude the main metabolic and endocrinological diseases), brain MRI, and abdominal ultrasound were performed. These examinations showed no alterations.

NGS panel for HI revealed a mutation in the *ABCC8* gene, a missense variant inherited from the father, which was associated with the condition of HI. The diagnostic process was continued with the performance of an ^18^F-DOPA PET/MRI, which revealed a diffuse form of pancreatic beta-cell hyperplasia. The octreotide dose was gradually increased to a maximum of 20 mcg/kg/day, with a progressive normalization of glucose levels. The patient was discharged at around two months of age.

## Discussion

Clinical presentation of the reported cases

Congenital HI typically manifests in the neonatal period (about 90%), and more rarely, the onset occurs in childhood or adolescence (about 10%) [9]. In the case of the first patient, persistent neonatal hypoglycemia, caused by the mutation in the *KCNJ11* gene, overlapped with neonatal sepsis, which could have complicated the diagnostic process; the second patient did not have any comorbidities, which made the signs of hypoglycemia more easily identifiable.

The clinical symptoms of hypoglycemia can be non-specific during the neonatal period. The clinical symptoms of hypoglycemia vary from non-specific and mild adrenergic symptoms (poor feeding, hunger, palpitations, and sweating) to life-threatening, neuroglycopenic symptoms (apnea, seizures, unconsciousness, lethargy, coma, and even death) [10,11]. These signs and symptoms are non-specific and do not always make hypoglycemia easily identifiable, especially if sepsis develops concurrently, as in the case of the first patient who presented with neurological involvement, including hypertonic seizures and tonic-clonic jerks.

The first patient had normal auxological parameters (adeguate for gestational age), while the second one had a birth weight at the upper limits. High birth weight is commonly reported as a clinical feature of congenital HI; nevertheless, the condition can also occur in infants with normal or low birth weight [12].

Pathophysiology of congenital HI

The increase in plasma glucose levels above approximately 85 mg/dL serves as a stimulus for insulin secretion by the pancreatic beta cells [13]. Glucose enters the beta cells via specific glucose transporters and undergoes metabolic processing, leading to the production of ATP. The rise in ATP/adenosine diphosphate ratio causes ATP-sensitive potassium channels to close, resulting in membrane depolarization. Depolarization opens voltage-gated calcium channels, allowing Ca²⁺ influx into the cell, and increased intracellular Ca²⁺ triggers the fusion of insulin-containing vesicles with the plasma membrane, releasing insulin into the bloodstream. Defects in any part of this pathway can impair insulin secretion and contribute to the development of hypoglycemia [13].

Genetic background and phenotype

According to the literature, genetic testing is recommended in infants and children with persistent or refractory hypoglycemia in whom acquired or transient forms of HI have been reasonably excluded [7]. Mutations in at least 15 different genes have been identified in the literature as potential causes of congenital HI. The diffuse form in most cases is inherited in an autosomal recessive manner and affects all the pancreatic islets, whereas the focal form is sporadic and is confined to a small region of the pancreas [10].

These mutations lead to a dysfunction of the corresponding protein products in beta cells and can result in the dysfunction of ion channels (channelopathies), enzymes involved in energy metabolism (metabolopathies), and transcription factors (transcriptionopathies). Ion channel disruptions, as observed in the case of our patients, typically cause the most severe phenotype [12]. The *KCNJ11* and *ABCC8* genes encode the Kir6.2 and SUR1 subunits, respectively, of the ATP-dependent potassium channel present on beta cells. Inactivating mutations lead to the closure of potassium channels, resulting in membrane depolarization and an increase in intracellular calcium levels. This triggers insulin release even when cellular energy levels are low, ultimately causing hypoglycemia [10,11].

Diagnostic approach to neonatal hypoglycemia and HI

In order to correctly identify the etiology of hypoglycemia, samples should be collected, if possible, during a spontaneous episode and before any treatment that could rapidly alter levels of beta-hydroxybutyrate, FFAs, cortisol, or insulin. The primary criterion for the diagnosis of HI is the finding of elevated insulin levels concomitant with hypoglycemia. For diagnostic evaluation, a blood sample should be drawn to determine serum glucose, insulin, and C-peptide levels. Since a single measurement may yield false negatives due to the pulsatile nature of insulin secretion, three sets of samples should be obtained at different times to ensure accuracy. Measuring C-peptide is essential because it is more stable than insulin and is often elevated in HI. If obtaining a venous blood sample during spontaneous hypoglycemia is not possible, the most useful method for determining the etiology of hypoglycemia disorders is a provocative fasting test [4].

In cases of suspected HI, the fasting test may be terminated when plasma glucose falls below 50 mg/dL, a threshold sufficiently low to trigger the metabolic and neuroendocrine responses necessary for diagnosis. At this point, administration of glucagon (1 mg intravenously, intramuscularly, or subcutaneously) permits assessment of the glycemic response; an exaggerated increase in plasma glucose (>30 mg/dL) is considered highly suggestive, if not pathognomonic, of HI [4].

If serum insulin is detectable at glucose levels below 50 mg/dL, HI is confirmed. Insulin has anabolic function and inhibits ketogenesis and lipolysis. Lipolysis occurring in adipose tissue leads to the release of glycerol and free fatty acids (FFAs) into the bloodstream, which replace glucose as the energy substrate at the skeletal and muscular level but not at the cerebral level. FFAs are also converted in the liver into beta-hydroxybutyrate and acetoacetic acid, which can be used as an energy source at the cerebral level. The measurement of beta-hydroxybutyrate, free fatty acids, and lactate during hypoglycemia provides important information to determine the etiology [14].

To guide the diagnosis, it is important to distinguish between ketotic and non-ketotic hypoglycemia. The concentration of ketone bodies can be evaluated in serum and/or urine. Reduced levels of ketone bodies define non-ketotic hypoglycemia and are found in cases of metabolic disorders (such as defects in gluconeogenesis, glucagon storage diseases, organic acidurias, mitochondrial diseases, and cortisol and growth hormone deficiencies), panhypopituitarism, and congenital HI [15].

We should suspect panhypopituitarism in neonates who present with hypoglycemia associated with midline defects (such as cleft palate or choanal atresia) or micropenis. Non-ketotic hypoglycemia without HI should raise suspicion of fatty acid oxidation disorders [16].

High levels of ketone bodies are found in cases of ketotic hypoglycemia, such as glycogenosis, growth hormone deficiency, and cortisol deficiency [4].

Management and therapeutic strategies

If oral feeding is not feasible, emergency treatment of hypoglycemia consists of an intravenous bolus of 2 mL/kg of 10% glucose. In some cases, the bolus may need to be repeated, but it is important to remember that glucose administration is a strong stimulus for insulin secretion.

Normoglycemia is typically established through continuous intravenous glucose infusion, initially administered at 6-8 mg/kg/min. In cases of HH, substantially higher infusion rates (>25 mg/kg/min) may be necessary to sustain euglycemia.

In emergency settings where patients are unable to tolerate oral feeding and/or intravenous access is difficult to establish, glucagon should be administered. In the short term, glucagon promotes glycogenolysis, gluconeogenesis, and lipolysis, leading to a rapid increase in plasma glucose levels within minutes of administration [8].

The recommended single dose is 0.5 to 1 mg, administered either intramuscularly or subcutaneously. When used at higher doses (exceeding 1 mg), glucagon may induce rebound hypoglycemia as a result of a paradoxical stimulation of insulin secretion [17].

The frequency and severity of hypoglycemic episodes may be mitigated by regular feeding with high-calorie, carbohydrate-rich meal [8]. In patients with HI, glucagon administration (0.03 mg/kg) typically elicits a rise in blood glucose levels. An increased requirement for carbohydrate intake (>8 mg/kg/min, with a reference range of 4-6 mg/kg/min) provides additional diagnostic support [12].

When the diagnosis of HI has been made, the next crucial step is to determine whether there is a response to diazoxide; this trial not only provides therapeutic benefit but also important information on the phenotype.

Diazoxide, a K⁺-ATP channel agonist, is the first-line pharmacological treatment for congenital HI. The K⁺-ATP channel, encoded by the *ABCC8* and *KCNJ11* genes, normally closes in response to elevated intracellular energy levels within the beta cell [12].

Inactivating mutations of the *ABCC8* gene, as in the case of the second patient, are associated with non-expression of the SUR1 subunit on the plasma membrane of beta cells, resulting in non-responsiveness to diazoxide.

The starting dosage of diazoxide is 5 mg/kg/day; in more severe cases - such as when a glucose infusion rate exceeds 10 mg/kg/min - a higher starting dose of 10 mg/kg/day may be appropriate. If the patient still presents hypoglycemia after two to three days at this dose, diazoxide should be gradually increased up to a maximum of 15 mg/kg/day. Once the child has been on a stable dose for five days, a supervised fasting test ("safety fast") should be conducted to assess whether diazoxide has effectively controlled the underlying hypoketotic hypoglycemia, the hallmark of congenital HI. Children who show a positive response to diazoxide can be safely discharged on treatment, with appropriate home glucose monitoring. In contrast, if the child does not respond, diazoxide should be discontinued, and hypoglycemia should be managed with intravenous dextrose while also initiating urgent genetic testing. Approximately 90% of children with diazoxide-unresponsive HI harbor mutations in the *ABCC8* or *KCNJ11* genes. A subset of these patients present with a focal form of HI, which can be cured surgically [18].

Through the implementation of ^18^F-L-DOPA imaging, which in recent years has played an important role in the management of patients with congenital HI, we excluded focal disease. For children with diffuse HI unresponsive to diazoxide - such as those described in our series - therapy with octreotide, a short-acting somatostatin analog, is indicated. Treatment is initiated subcutaneously at 15 mcg/kg/day, administered in three divided doses, and can be escalated to a maximum of 30 mcg/kg/day.

Following the second year of life, therapy is often transitioned to a long-acting somatostatin analog (lanreotide), which allows for subcutaneous administration every six weeks. Sirolimus and exendin-(9-39) are emerging therapeutic agents with potential future applications. Sirolimus, by inhibiting the mammalian target of rapamycin pathway, reduces beta cell growth and insulin secretion. Exendin-(9-39) acts as an antagonist of the glucagon-like peptide 1 receptor [12].

## Conclusions

Both clinical cases demonstrate the importance of considering the diagnosis of congenital HI in the presence of persistent hypoglycemia and indicative clinical signs, both in conditions of clinical well-being and in confounding situations such as sepsis. Early recognition and timely treatment are crucial to prevent even severe complications of hypoglycemia with permanent outcomes. Molecular diagnosis makes it possible to predict the response to medical treatment and to assess the possibility of a focal form of congenital HI that would benefit from surgery; therefore, genetic analysis should be carried out as quickly as possible.
